# Label-Free Imaging of Lipid Droplets in Prostate Cells Using Stimulated Raman Scattering Microscopy and Multivariate Analysis

**DOI:** 10.1021/acs.analchem.2c00236

**Published:** 2022-06-14

**Authors:** Ewan W. Hislop, William J. Tipping, Karen Faulds, Duncan Graham

**Affiliations:** Centre for Molecular Nanometrology, WestCHEM, Department of Pure and Applied Chemistry, Technology and Innovation Centre, University of Strathclyde, Glasgow G1 1RD, U.K.

## Abstract

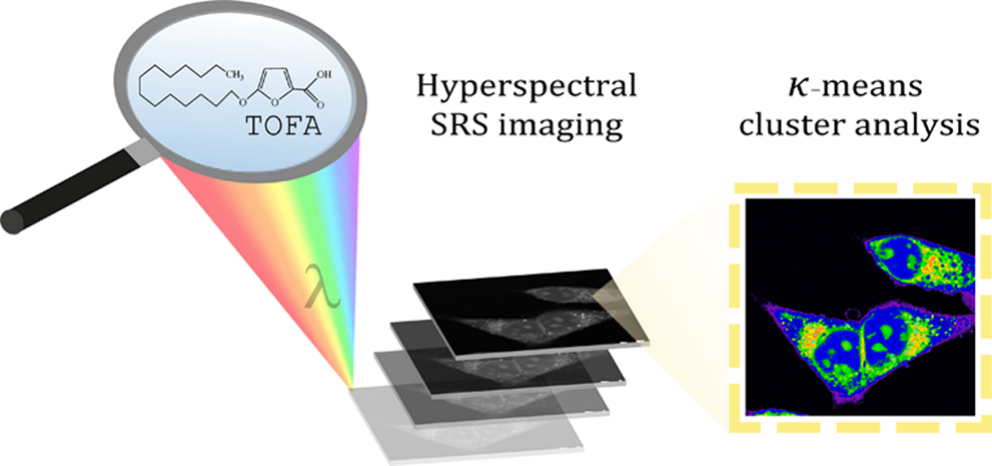

Hyperspectral stimulated Raman scattering (SRS) microscopy is a powerful imaging modality for the analysis of biological systems. Here, we report the application of *k*-means cluster analysis (KMCA) of multi-wavelength SRS images in the high-wavenumber region of the Raman spectrum as a robust and reliable method for the segmentation of cellular organelles based on the intrinsic SRS spectrum. KMCA has been applied to the study of the endogenous lipid biochemistry of prostate cancer and prostate healthy cell models, while the corresponding SRS spectrum of the lipid droplet (LD) cluster enabled direct comparison of their composition. The application of KMCA in visualizing the LD content of prostate cell models following the inhibition of de novo lipid synthesis (DNL) using the acetyl-coA carboxylase inhibitor, 5-(tetradecyloxy)-2-furoic acid (TOFA), is demonstrated. This method identified a reliance of prostate cancer cell models upon DNL for metabolic requirements, with a significant reduction in the cellular LD content after treatment with TOFA, which was not observed in normal prostate cell models. SRS imaging combined with KMCA is a robust method for investigating drug–cell interactions in a label-free manner.

Innovations in diagnostics, molecular characterization, and treatment of prostate cancer (PCa) have improved clinical outcomes; however, among men, it still remains the most frequently diagnosed cancer and the second leading cause of death worldwide.^1^ Despite numerous primary treatment strategies chiefly targeting androgen,^2^ in advanced PCa, adaptation to therapy often leads to disease progression and castration-resistant phenotypes. Preclinical models have identified complex resistance mechanisms such as androgen receptor (AR) amplification and mutations,^3^ while others are associated with altered metabolic pathways.^4^

The upregulation of de novo lipid synthesis (DNL) is cited as a biomarker of aggressive PCa disease,^5^ while inhibiting DNL has therapeutic potential for PCa treatment.^6^ The overexpression of several key enzymes (e.g., acetyl-coA carboxylase, ACC, and fatty acid synthase, FAS) involved in DNL (Figure 1A) stimulates energy fluxes to meet metabolic lipid demands.^7^ One such example is 5-(tetradecyloxy)-2-furoic acid (TOFA) which inhibits the rate-limiting enzyme, ACC responsible for the conversion of acetyl-CoA into malonyl-CoA (Figure 1B). TOFA has been shown to suppress proliferation and induce apoptosis in the colon cancer cell lines, HCT-8 and HCT-15.^8^ Investigating the regulation of lipid droplets (LDs) could be targeted for drug development or their increased biogenesis inspected as potential biomarkers for the disease.^9^

**Figure 1:**
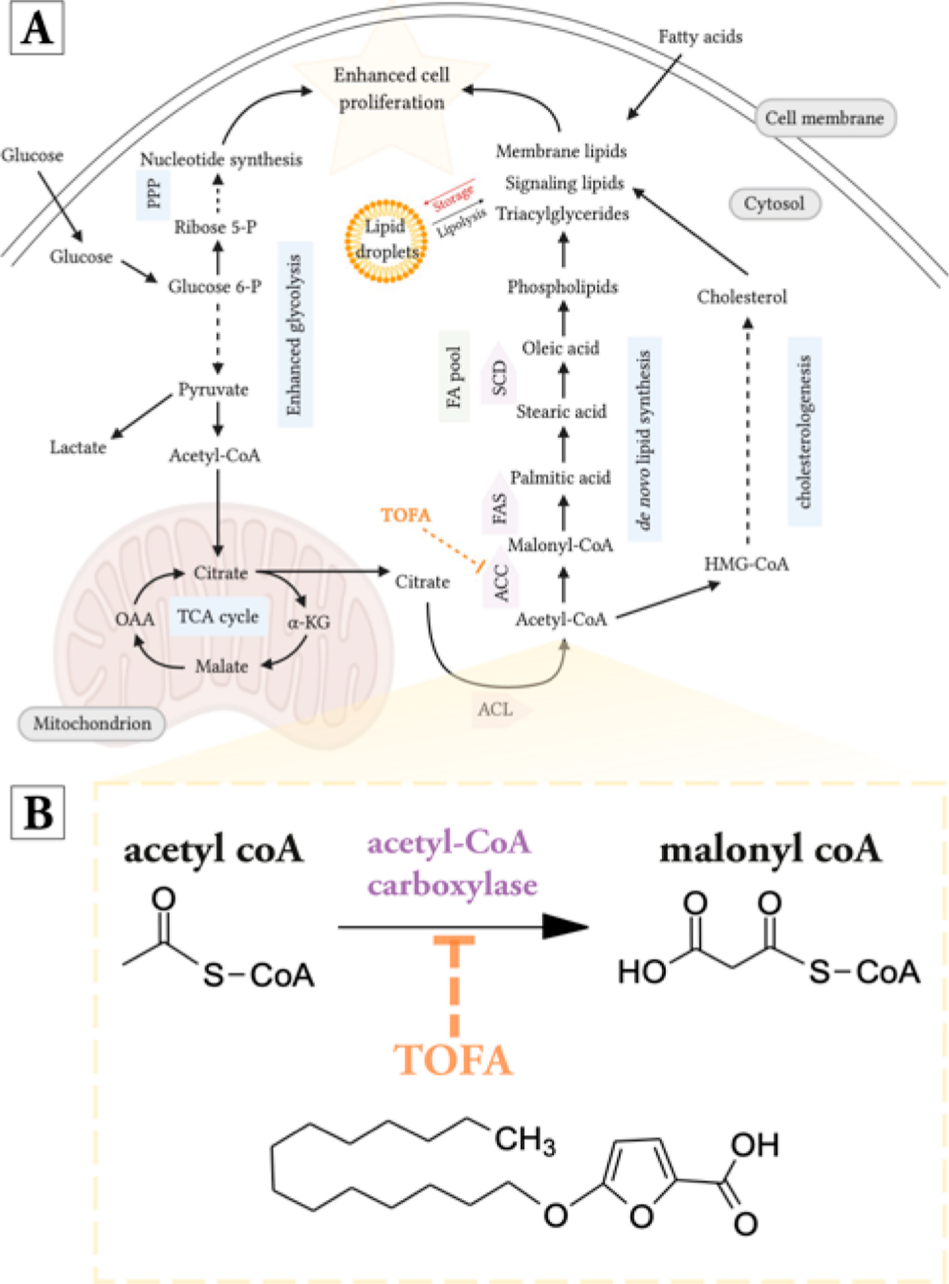
Overview of the lipid biosynthesis pathway. (A) Upregulated metabolic processes contribute to cancer growth and proliferation during malignancy. Solid arrows indicate single reaction processes and dotted arrows indicate processes with multiple reactions. αKG, α-ketoglutarate; ACC, acetyl-CoA carboxylase; FAS, fatty acid synthase; glucose 6-P, glucose 6-phosphate; HMG-CoA, 3-hydroxy-3-methyl-glutaryl-coenzyme A; OAA, oxaloacetate; PPP, pentose phosphate pathway; ribose 5-P, ribose 5-phosphate; and SCD, stearoyl-CoA desaturase. (B) Investigating the effect of TOFA in cell lines associated with PCa malignancy by targeting ACC enzyme in DNL and imaging associated phenotypes by SRS. The small-molecule TOFA targets the initial step in the pathway, inhibiting the conversion of acetyl-CoA into malonyl-CoA.

Optical imaging of cellular LDs in prostate cancer cells has been demonstrated using brightfield^10^ and fluorescence microscopy^11^ in combination with hydrophobic stains that provide contrast for imaging; typical examples include Nile Red, Oil Red O, and BODIPY dyes.^12^ However, molecular stains intrinsically disrupt the LD composition and may perturb the biophysical properties of the LD membrane.^13^ As such, label-free imaging modalities provide a clear advantage to the use of imaging stains. Infrared spectroscopy has been used to visualize the lipid content in prostate cells, although the spatial resolution restricted the analysis to whole-cells.^14^ Raman spectroscopy is a preferred optical imaging tool because it can provide label-free visualization of cellular samples under biocompatible imaging conditions. The technique has been applied to various aspects of prostate cancer including, biopsy analysis, tissue resection imaging, and biofluid analysis.^15^ Notably, ratiometric Raman imaging has been used to characterize the impact of TOFA treatment upon the global lipid pool in prostate cell models using the CH_2_ symmetric stretch at 2851 cm^–1^ as a marker.^16^ However, the resolution for the Raman mapping experiments was insufficient to investigate the impact of TOFA treatment at the LD level.

The development of stimulated Raman scattering (SRS) microscopy has brought about improvements in the spatial resolution, 3D imaging capability and temporal analysis. SRS has enabled studies of lipid metabolism in a variety of cellular models including, brain,^17^ pancreatic,^18^ and prostate cancers,^19^ and in organisms including mice^20^ and drosophila flies.^21^ Hyperspectral SRS imaging facilitates biochemical characterization based directly on the SRS spectrum. Several recent reports have applied advanced chemometric analysis techniques to hyperspectral SRS imaging data to extricate the underlying biochemical information. Of these, spectral phasor analysis has proven fruitful for cell segmentation,^22^ cytometry applications,^23^ and investigating drug–cell interactions.^24^ An alternative chemometric analysis tool for Raman spectral data is *k*-means cluster analysis (KMCA) which has been widely applied in Raman spectroscopic analysis of cells,^25^ intracellular nanoparticles,^26^ and ex vivo tissue samples.^27^ Perhaps surprisingly, KMCA is not widely reported for hyperspectral SRS data in the same way; two notable examples include compositional analysis of lipid storage in *Caenorhabditis elegans* worms^28^ and the analysis of meibum secretions as a thin film.^29^

Herein, we report KMCA analysis of SRS spectral data sets as a robust and reliable methodology for cellular segmentation and spectral analysis. We report the first SRS imaging across the high-wavenumber region of the Raman spectrum (2800–3100 cm^–1^) and KMCA for the segmentation of intracellular LDs. This method provided a label-free approach to assess the composition of LDs and the impact of TOFA treatment in a panel of prostate cell models. The analysis of TOFA treatment in prostate cancer cell models indicated a strong reduction in the LD number and distribution compared to the non-cancerous, PNT2 cells. The associated SRS spectra of the LDs identified a reduction in unsaturated lipid content in the TOFA-treated cells compared to the untreated cells; this effect is not observed in non-cancerous, PNT2 cells. These results indicate a clear potential for SRS spectral imaging and KMCA for investigating drug–cell interactions with chemical specificity, subcellular resolution, and in a label-free manner.

## Results and Discussion

**Figure 2:**
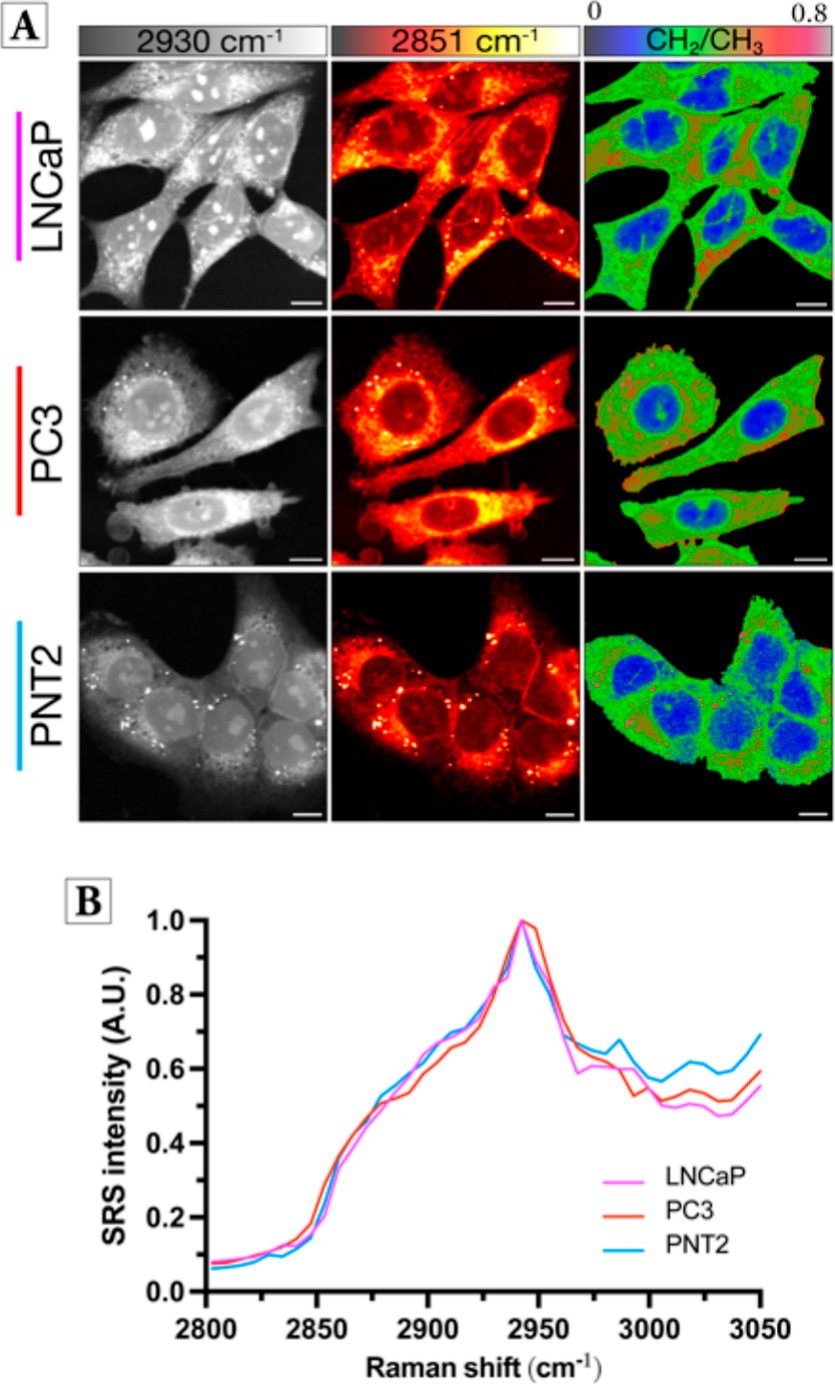
Characterization of prostate cell models using SRS microscopy. (A) SRS images were acquired from live LNCaP, PC3, and PNT2 cells at 2930 cm^–1^ (CH_3_ symmetric stretch, proteins) and 2851 cm^–1^ (CH_2_ symmetric stretch, lipids). A ratiometric image of the CH_2_/CH_3_ is presented. The background (non-cell areas) has been removed (see the Experimental Section for details). SRS images acquired at 512 × 512 pixels with a 24 μs/pixel dwell time and false colors assigned to different detection wavenumbers. Scale bars: 10 μm. (B) Mean SRS spectra acquired from the cells presented in (A). Sequential SRS images were acquired across the range 2800–3050 cm^–1^ (∼0.4 nm step, ∼6 cm^–1^, 40 images) and the mean SRS spectrum determined across all the cells in the image. The spectra are normalized between 0 and 1.

To evaluate the pharmacological effects of TOFA in metastatic PCa, we selected two cell lines with different subtypes, including androgen-dependent (LNCaP) and castration-resistant prostate cancer cell models (PC3). Molecular differences between the two cell lines are considered to be accountable for the aggressiveness or progression of the disease.^30^ Retaining a well-differentiated morphology observed in luminal cells of the glandular prostate, epithelial PNT2 cells were selected to enable comparison between normal and malignant prostate cell models. Label-free SRS imaging was first utilized to characterize the global protein and lipid distribution in all three cell lines (Figure 2A).

The frequency difference between the pump (tunable between 700 and 960 nm) and Stokes beams (1031 nm) was tuned to be resonant with the endogenous biomolecules of interest.^31^ Image acquisition at 2930 cm^–1^ (CH_3_ symmetric stretch) was indicative of protein signals that were visualized in the cytoplasm, nuclei, and nucleoli in each cell line. Images acquired at 2851 cm^–1^ (CH_2_ symmetric stretch) identified the localization of endogenous lipid biomolecules predominately found in the cell cytoplasm and located within the LDs. Ratiometric analysis revealed the nuclear region of the cells with a relatively low CH_2_/CH_3_ ratio, while LDs were detected within all cell lines and appear as bright spots in the cell cytoplasm with a CH_2_/CH_3_ ratio that is typically greater than 0.8. Next, SRS images were acquired across the high-wavenumber region in a wavelength scanning experiment, and the average SRS spectrum was determined across the cell populations (Figure 2B). The normalized spectra show characteristic peaks at 2930 cm^–1^ (proteins) and 3015 cm^–1^ (=CH) indicative of triacylglycerols (TAGs). However, when averaging the SRS spectrum across the whole cell, the intensity at 2851 cm^–1^ appears relatively low, which is a likely reflection of the fact that in each cell line, the number of LDs is low. Altogether, these data confirmed the applicability of SRS microscopy for the label-free detection of the cellular protein and lipid content and the biochemical characterization based on the SRS spectrum. To improve the accuracy in the analysis of cellular LDs, we elected to apply multivariate analysis of the SRS spectral data. KMCA has been previously used for segmentation of lipid compartments in *C. elegans* worms^28^ and meibum secretions,^29^ yet it has not been used to investigate the biochemistry of LDs in mammalian cells and the impact of drug treatment upon lipid metabolism. KMCA partitions the data based on spectral similarity with further information reported previously.^32^ Initially, wavelength scanning SRS imaging was performed across the high-wavenumber region (2800–3050 cm^–1^) by image acquisition and subsequent retuning of the pump beam by ∼0.4 nm (∼6 cm^–1^, 40 individual images) between image frames. The image stack was combined, and an average intensity projection was created, which maps the average pixel intensity at each location across the image. Next, KMCA was performed on the spectral data set using a plug-in for ImageJ, which was developed for the analysis of fluorescent images.^33^ KMCA has been widely applied to the study of cells and tissues using spontaneous Raman scattering, whereby spectra are clustered based on the similarity of the spectral profile. As such, the clustering reflects the molecular information contained within the sample and the clusters can be mapped back onto the original SRS image stack to create false-color segmented images of the spatial distribution of each cluster, thereby identifying regions with similar biochemical signatures.

**Figure 3:**
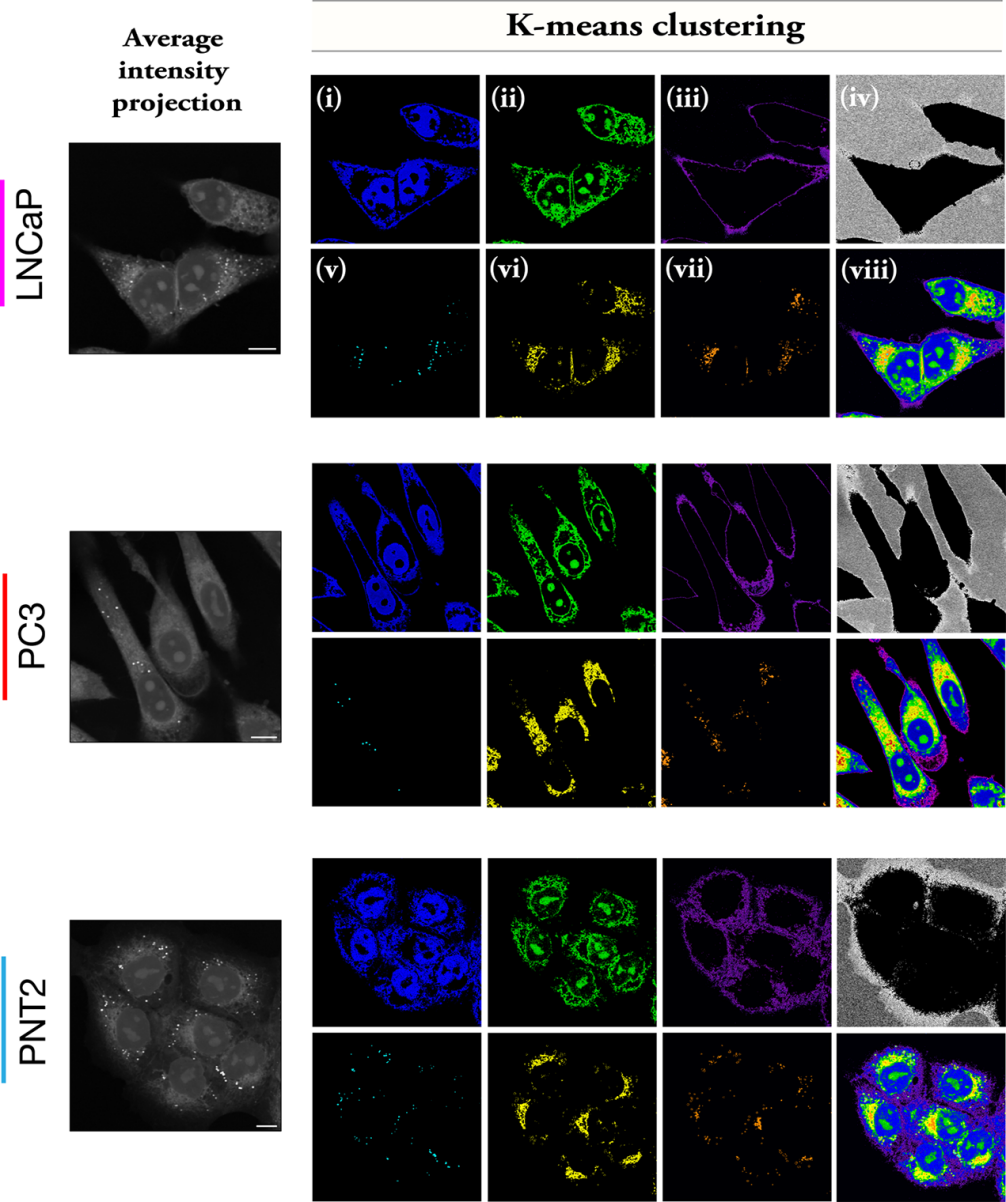
Cellular segmentation using KMCA. SRS image stacks were acquired across the region 2800–3050 cm^–1^, from which the average intensity projection was created (scale bars: 10 μm). KMCA resulted in seven clusters (i–vii) for each cell line based on the corresponding SRS spectra at each pixel location. A composite image is presented for each cell line (viii).

In the first instance, we acquired SRS image stacks across the range 2800–3050 cm^–1^ for each cell line. KMCA was applied to the resulting data sets to enable segmentation of individual intracellular features based directly on the corresponding SRS spectra. Figure 3 presents the KMCA of populations of LNCaP, PC3, and PNT2 cells. In each case, the KMCA was presented as false-color images, thereby segmenting the cell into seven clusters based on the SRS spectral features. In each case, it was possible to create segmented images corresponding to regions of high protein content including (i) the nucleus and cytoplasm (blue), (ii) nucleoli (green), and (iii) cell boundary (purple). The clustering of the nucleus and cytoplasm regions in (i) suggest that protein signal is the likely discriminator for this cluster because cellular DNA is largely confined to the nucleus. This result is also in agreement with a recent report that segmented protein and lipid rich regions in ovarian cancer cells using least absolute shrinkage and selection operator (LASSO) spectral unmixing.^34^ It is interesting to note that the nucleoli cluster differently to the rest of the nucleus in each of the three cell lines, and may be a reflection of the different levels of DNA, RNA, and protein content across the nucleus as a whole. Areas corresponding to the non-cell background were clustered in (iv). Lipid-rich regions were clustered into (v) LDs (cyan), while (vi) and (vii) localize lipid-rich regions in the cytoplasm that may correspond to the endoplasmic reticulum and mitochondria,^26^ although this has not been validated. It is interesting to note that similar cellular segmentation had been retrieved by spontaneous Raman imaging and KMCA of A549 cells (human lung carcinoma cell line),^26,35^ albeit with a reduced spatial resolution when compared to SRS microscopy. A merged image of the clusters is also presented in (viii) to identify the relative locations of each cluster across the cells. To that end, KMCA was shown to be suitable for analyzing cellular LDs across the cell populations in a way that is not readily achievable in our previous analysis using ratiometric Raman imaging.^16^

**Figure 4:**
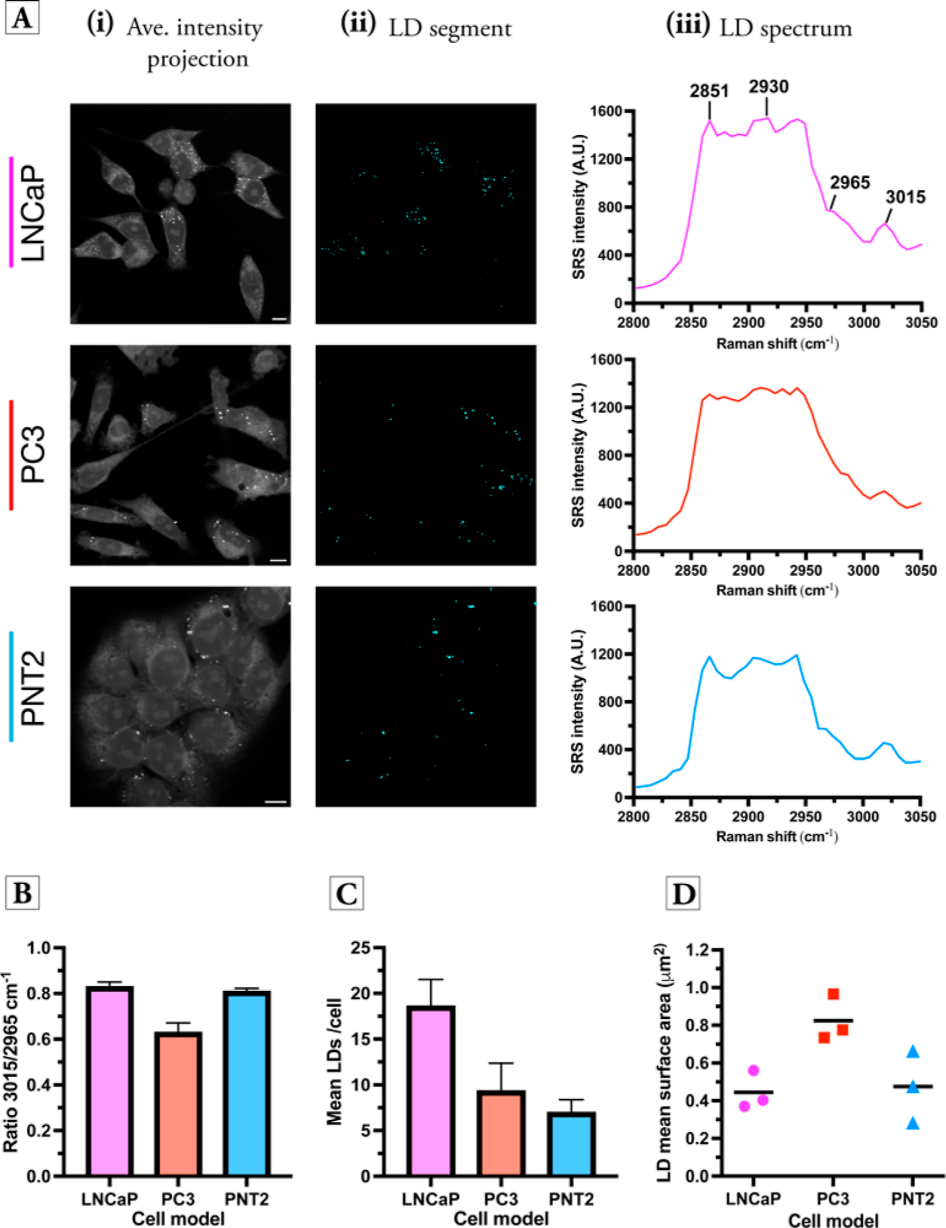
Investigating cellular LDs in prostate cell lines using KMCA. (A) An average intensity projection from SRS image sweeps for each cell line is presented in (i) alongside the LD content of the cells identified using KMCA in (ii). The average LD spectrum is plotted for each cell line using the segmented LD image as a marker to determine the mean SRS spectrum (iii). Peak annotations: 2851 cm^–1^ (CH_2_ symmetric stretch), 2930 cm^–1^ (CH_3_ symmetric stretch), 2965 cm^–1^ (cholesteryl esters), and 3015 cm^–1^ (=CH unsaturated lipid). Scale bars: 10 μm. (B) Quantification of the ratio 3015/2965 cm^–1^ in each cell line. Data represent mean ratio from three replicate images, error bars + S.D. (C) Quantification of the mean number of LDs per cell [identified in (A)]. In each case, >35 cells were analyzed across three biological repeats. (D) Quantification of the mean surface area of cellular LDs identified in (A). Data points represent the mean surface area of all LDs in all cells from three biological replicates.

A schematic workflow for the data acquisition and KMCA analysis is provided in Figure S1. Having created segmented images of the cell populations, it was possible to assess the LD distribution and SRS spectral characteristics of LDs in the three cell lines tested. Figure 4A presents the segmented images of the LDs via KMCA of the hyperspectral SRS image stacks in each cell line. Using the segmented images as a marker, it was then possible to characterize each cluster based on the mean SRS spectrum. The cluster associated with LDs presented peaks at 2851 cm^–1^ (CH_2_ symmetric stretch), 2965 cm^–1^ (cholesteryl esters), and 3015 cm^–1^ (=CH, unsaturated lipids) which are indicative of cellular lipid species.^36^ Interestingly, the ratio of 2851/2930 cm^–1^ is ∼1, which was consistent with our ratiometric analysis in Figure 2A. Furthermore, the significant SRS signal detected at 3015 and 2965 cm^–1^ is indicative of unsaturated lipids (TAGs) and cholesterol esters (CEs), respectively.^37^ The average SRS spectra of cellular LDs suggest that TAGs are a major component of LDs in prostate cells due to the spectral similarity to a previous report that identified TAGs in ovarian cancer cells.^34^ We assessed the ratio 3015/2965 cm^–1^ (TAGs/CEs) for the three prostate cell lines (Figure 4B). These data indicated that in the LNCaP and PNT2 cells, the LDs contain large quantities of TAGs with 3015/2965 cm^–1^ ratios >0.75, whereas in the case of PC3 cells, the ratio 3015/2965 cm^–1^ is ∼0.6, which indicated greater levels of CEs. These findings are supported by a previous analysis using hyperspectral SRS imaging of lipid mixtures of 100% TAGs (3015/2965 cm^–1^ = 0.75) and 100% CEs (3015/2965 cm^–1^ = 0.29),^38^ while fingerprint Raman spectroscopy previously identified elevated levels of CEs in PC3 cells based on the intensity of the peak at 702 cm^–1^ (cholesterol ring stretch), which was reduced in LNCaP cells at low passage numbers.^19^ Our findings indicated that SRS imaging with KMCA is capable of investigating the basal LD composition in cellular models in a robust way.

Furthermore, we determined the number of LDs per cell in each case; LNCaP cells had more LDs per cell compared to PC3 cells, and the cancerous cell lines had more LDs than the non-cancerous, PNT2 cells (Figure 4C). In addition, we determined the LD mean surface area in each cell line (Figure 4D). Interestingly, PC3 cells presented a greater LD surface area than the LNCaP and PNT2 cells. These data confirm the higher basal levels of lipid in PCa cell models compared to healthy cell models, and that there was a significant difference in the composition and morphology of LDs among the three cell lines. Altogether, these data represent the first investigation into cellular LDs based on KMCA segmentation which is achieved without the use of hydrophobic dyes for contrast. To validate these findings, we performed ratiometric Raman mapping of live cells using 532 nm excitation and a 1 μm pixel size. The average Raman spectrum from all the pixels within the ratiometric Raman image demonstrated a lower lipid content in PNT2 cells compared to the cancerous LNCaP and PC3 cells (Figure S2i). Additionally, we quantified the mean intensity of the SRS signal at 2851 cm^–1^ across the whole cell, which confirmed the lower lipid content associated with PNT2 cells (Figure S2ii). Lastly, PNT2 cells also presented fewer LDs per cell (Figure S2iii) consistent with our KMCA result in Figure 4B.

**Figure 5:**
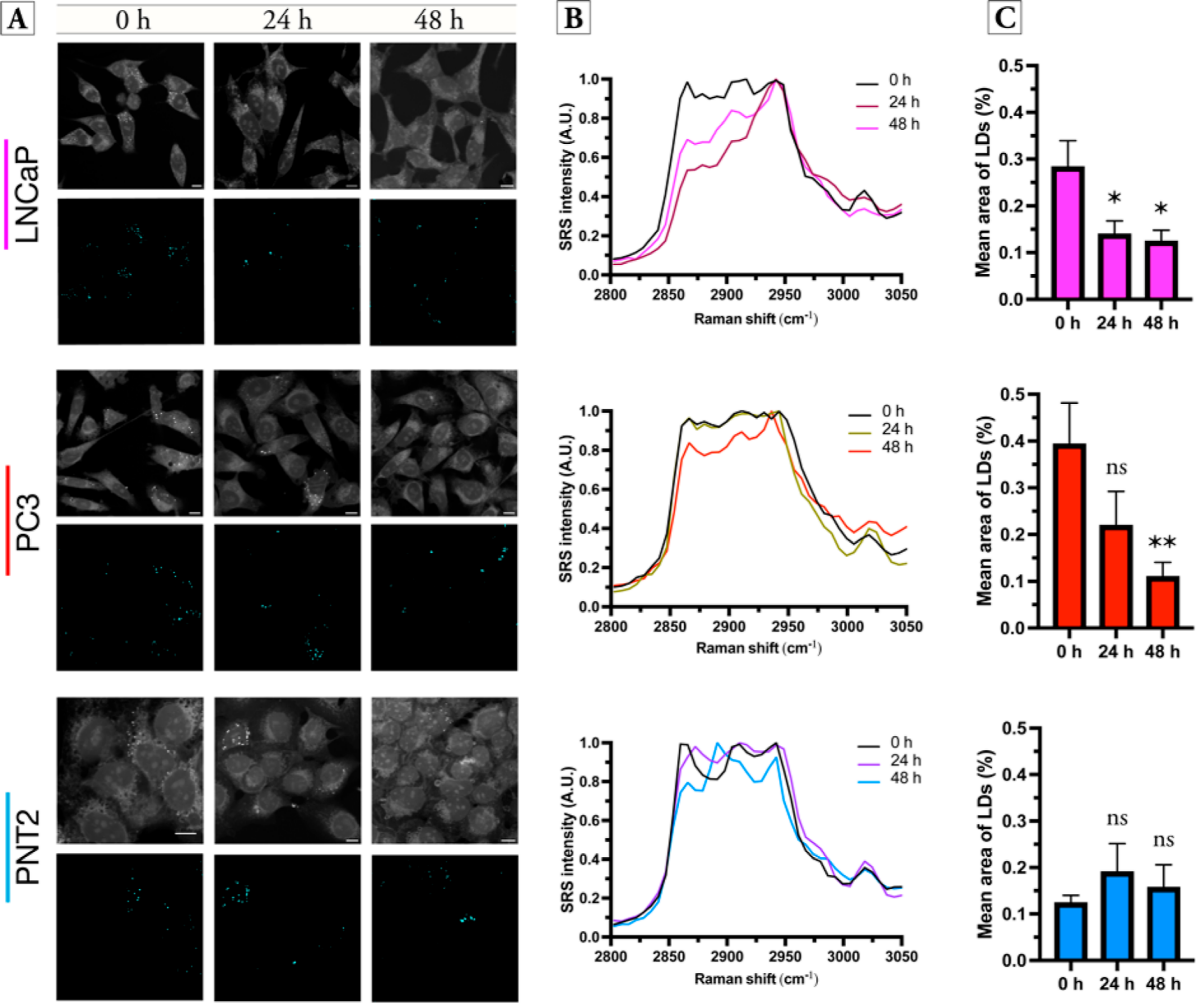
Investigating the effect of TOFA treatment upon prostate cell lines. Prostate cell lines were treated with TOFA (5 μM) for the indicated times, and SRS images acquired across the range 2800–3050 cm^–1^. (A) An average intensity projection across the field-of-view is provided (scale bars: 10 μM). KMCA was used to segment the LDs in each cell population at each timepoint. (B) Mean SRS spectrum from segmented LDs. (C) Analysis of the mean % area of LDs in the cell population. Data represent the mean ± SEM from three biological repeats with approximately 10 cells per field-of-view. Student’s *t*-test was employed to assess significance between the mean area of LDs (%) in cells exposed to TOFA (*P ≤ 0.05, **P ≤ 0.01).

We next investigated the effect of TOFA treatment upon cellular LDs using SRS imaging and KMCA segmentation. Each cell line was incubated with TOFA (5 μM for 24 h or 48 h) or DMSO (0.1% v/v) as a control. SRS images were acquired across the high wavenumber region as described previously. Figure 5 presents the KMCA results based on the LD cluster in each cell line following 24 and 48 h treatment with TOFA. In the malignant LNCaP and PC3 cells, TOFA treatment resulted in an overall reduction in LDs per cell (Figure 5A), indicating that these cell lines are dependent upon DNL to meet their energy requirements. In the case of LNCaP cells, the effect of TOFA treatment is most pronounced and KMCA of LNCaP cells exposed to TOFA for 24 and 48 h showed that the cells did not contain any cellular LDs, while the DMSO-treated LNCaP cells presented numerous cytoplasmic LDs. However, KMCA did identify a separate cluster in the TOFA-treated LNCaP cells (Figure 5A) that had a lower 2851/2930 cm^–1^ ratio (lipid/protein) compared to the LD cluster in the control LNCaP cells (Figure 5B), and this cluster was shown to have a significantly lower cellular % area compared to control cells (Figure 5C). This observation suggests that LNCaP cells metabolize their LDs as an energy source upon ACC inhibition. Similarly, PC3 cells showed a reduction in cellular LDs following TOFA treatment, although the average SRS spectra show that some signal is detected at 3015 cm^–1^ after 48 h TOFA treatment. PC3 cells are reported to display a more aggressive cancer phenotype when compared to LNCaP cells,^39^ and the ability of PC3 cells to circumvent DNL inhibition may provide an explanation for this. Furthermore, while imaging PC3 cells, we observed a subset of the cell population with significantly elevated LDs, and notably these cells appeared to be multinucleated. Figure S3 presents the average intensity projection and KMCA segmentation of a multinucleated PC3 cell. Notably, a significant number of LDs are detected in the perinuclear region and throughout the cell cytoplasm, and the clustering reveals multiple nuclei within the confines of the cell. Following KMCA, the corresponding mean SRS spectra revealed elevated levels of TAGs when compared to the LDs associated with mononucleated cells (Figure S3). Multinucleation is a result of mitotic slippage, a process where cells exit mitosis and enter interphase without going through chromosome segregation and cytokinesis, resulting in cells that are multinucleated.^40^ Despite ACC inhibition, the multinucleated cell presented significant lipid accumulation (Figure S3), that is, far greater than the basal levels observed in the mononucleated cell in our earlier analysis (Figure 4).

Lastly, in the case of PNT2 cells, TOFA treatment at 5 μM did not result in a reduction in the cellular LDs (Figure 5). Furthermore, the mean SRS spectra of the LDs in PNT2 cells showed no overall change in the intensity of the 3015 cm^–1^ (TAGs) and no overall change in the % area LDs per cell. In most normal mammalian tissues, the uptake of exogenous lipid is preferred for the synthesis of new structural lipids, whereas DNL is usually supressed.^41^ In contrast, DNL is elevated in cancer cells,^42^ which supports the observation that TOFA treatment reduced the lipid content in the cancerous cell lines used in this study. To validate the KMCA results on the effect of TOFA treatment in prostate cell models, SRS images were acquired at 2851 cm^–1^ and the signal intensity was quantified for each cell line (Figures S4–S6). These data indicated a reduction in the lipid content across the cancerous cell lines, while no significant difference was observed in the healthy PNT2 cells. An advantage of using KMCA is that the effect of TOFA treatment upon the lipid biochemistry is revealed rather than the requirement for threshold analysis of SRS signal intensity which can be subjective. As further validation of the KMCA experiments, the effect of TOFA treatment (in the range 5–20 μM) upon each of the cell lines was also investigated using ratiometric Raman mapping, which revealed a dose-dependent reduction in the 2851 cm^–1^ signal in the cancerous cell lines (Figure S7). A much weaker effect was observed in the PNT2 cells at a concentration of 5 μM when the average Raman spectrum was determined across the whole cell. Therefore, KMCA has enabled direct investigation of TOFA treatment at the LD level, which was not achieved using ratiometric Raman imaging, and identified changes in the biochemical profile of the LDs as a result. The improved spatial resolution of SRS imaging compared to spontaneous Raman spectroscopy also represents and advantage for investigating cellular LDs in biological systems. Further advantages of the current method are label-free detection in a non-destructive manner, which are limiting factors in both fluorescence and mass spectrometry (MS)-based imaging approaches. As an unsupervised multivariate technique, KMCA has enabled a robust delineation of cellular LDs without the requirement of user-defined segmentation, although the mean SRS spectrum that is generated from our analysis may underrepresent the subtle differences in biological composition across the sample. To overcome this limitation, a pairwise approach of KMCA followed by principal component analysis (PCA) could be an effective strategy going forward^25^ to extricate the subtle compositional changes of cellular LDs during drug treatment.

## Conclusions

SRS microscopy in combination with KMCA has enabled the direct segmentation of prostate cell lines without the use of fluorescent stains for contrast. The direct detection of LDs within prostate cell models was achieved using SRS imaging across the high-wavenumber region of the Raman spectrum, and retrieval of the corresponding SRS spectra was made possible using KMCA. The size and distribution of cellular LDs was visualized with high spatial resolution and chemical specificity to enable direct comparison across the panel of cells. Furthermore, the inhibition of ACC using the small molecule inhibitor, TOFA, resulted in a reduction in cellular LDs in prostate cancer cell models, while in normal prostate cell models, no reduction in LDs was observed. The data obtained in PNT2 cells indicated that they are not reliant upon DNL to satisfy their energy requirements. KMCA also identified the clear presence of multinucleation in PC3 cells following TOFA treatment alongside a significant increase in cellular LDs in a multinucleated cell, despite ACC inhibition. The formation of multinucleated cells has been linked as a potential chemoresistance mechanism, and the data presented here indicated that DNL inhibition may promote mitotic slippage toward cellular multinucleation, although further research would be required to validate this. Furthermore, post-processing methods including multivariate curve resolution and PCA, which have been shown to be effective methods to enhance discrimination of the spectra following KMCA could be explored to facilitate the analysis of the clustered SRS spectra. The application of KMCA to investigate other cellular processes is envisaged. Given the wide variety of bioorthogonal Raman labels targeted to the cell-silent region,^31^ KMCA could facilitate the analysis and robust segmentation of multiplex labels in the same cellular sample.

## Experimental Section

### Reagents and Chemicals

TOFA was obtained from Merck (>98%) and used as supplied. A 20 mM stock solution was prepared in anhydrous DMSO.

### Cell Culture

PC3 (human prostate adenocarcinoma) cells and PNT2 (human normal immortalized prostate epithelium) cells were gifted from the Strathclyde Institute of Pharmacy and Biomedical Sciences (Glasgow) as a subculture from a stock received from the European Collection of Authenticated Cell Cultures (ECACC). LNCaP (Lymph Node Carcinoma of the Prostate) cells were gifted by Professor Hing Leung from the Beatson Institute of Cancer Sciences (Glasgow). PC3 cells were maintained in Dulbecco’s modified Eagle’s medium (DMEM, Sigma-Aldrich) supplemented with 2 mM l-glutamine, while prostate cancer cell lines LNCaP and PNT2 were cultivated in Roswell Park Memorial Institute Medium (RPMI). Both formulations of media were supplemented with 10% (v/v) heat-inactivated foetal bovine serum (FBS), 1% (v/v) penicillin–streptomycin (10 000 units/mL), and 1% amphotericin B (Life Technologies, Thermo Fisher). Each cell line was propagated in T-175 Falcon flasks at 37 °C within a humidified incubator containing 5% CO_2_, where exponential growth was sustained between 0.25 and 1.5 × 10^6^ cells/mL. Routine subculture was performed at ca. 80% confluency and the passage number was kept below 20. For SRS experiments, cell cultures were passaged and harvested every 3–4 days.

### Stimulated Raman Scattering Microscopy

An integrated laser system (picoEMERALD S, Applied Physics & Electronics, Inc.) was employed to generate two synchronized laser beams at 80 MHz repetition rate. A Stokes beam (1031.4 nm, 2 ps pulse width) was intensity modulated by an electro-optic-modulator (EOM) with >90% modulation depth, and a tunable pump beam [700–960 nm, 2 ps pulse width, <1 nm (∼10 cm^–1^) spectral bandwidth] was produced by a built-in optical parametric oscillator (OPO). For SRS measurements, the Stokes beam was modulated with a 20 MHz EOM. The pump and Stokes beams were spatially and temporally overlapped via a series of dichroic mirrors and a delay stage inside the laser system, paired to an inverted laser-scanning microscope (Leica TCS SP8, Leica Microsystems), where the beams were focused onto the sample by a 40× objective (HC PL IRAPO 40×, N.A. 1.10 water immersion lens). Forward scattered light was collected by a S1 N.A. 1.4 condenser lens (Leica Microsystems). The Stokes light was removed and the pump beam intensity measured by a silicon photodiode connected to a lock-in amplifier (Applied Physics & Electronics, Inc.). The lock-in amplifier signal was fed into the Leica Microsystems SP8 microscope. The laser powers measured after the objective lens were in the range 10–30 mW for the pump beam only, 10–50 mW for the Stokes beam only and both synchronized beams at 20–70 mW. SRS images were acquired with a 9.75–48 μs pixel dwell time over a 512 × 512 or 1024 × 1024 frame at 12-bit image depth and recorded using Leica application suite (LAS X) software. Polystyrene beads (∼1 μm) were used to calibrate the multimodal setup through the detection of SRS signal at 3050 cm^–1^. All images were captured using the aforementioned custom-built multi-photon confocal microscope at the University of Strathclyde.

#### SRS Cell Imaging

Harvested cells (PC3, LNCaP, and PNT2) were seeded with a density of 1 × 10^6^ cells onto high precision coverslips (#1.5H thickness, 22 × 22 mm, Thorlabs) in six-well culture dishes (Costar) with 2 mL of their respective media and incubated at 37 °C and 5% CO_2_ for 24 h prior to the treatment. From a 20 mM stock solution in DMSO, cells were treated with TOFA (5 μM) in media and incubated at 37 °C and 5% CO_2_ for the indicated time. Control cells were concomitantly treated with DMSO at an equivalent rate in the respective media (0.05% DMSO v/v). Prior to imaging, cell culture plates were washed with PBS (2 × 2 mL) and 4% paraformaldehyde was added (2 mL for 15 min). Following fixation, the coverslips were washed with PBS (2 × 2 mL) and mounted on glass microscope slides with a PBS boundary between the glass layers using a method as previously described in Fu et al.^43^ A typical field-of-view was 150 × 150 μm containing a minimum of 5–10 cells.

### Data Processing

#### SRS Images

False-color assignments, scale bars, and image overlays were added using ImageJ software. Consistent brightness/contrast settings were applied when comparing average intensity projections in all figures. Ratiometric images were created by dividing the CH_2_ image by the CH_3_ image (2851/2930 cm^–1^), then multiplying with a threshold CH_3_ image to locate the cell areas, and the non-cell areas were set to 0. The extracellular background was removed using the Image Calculator function by multiplying the ratio image by the CH_3_ threshold mask using ImageJ. The subsequent image was scaled between 0 and 0.8 units and presented in Rainbow RGB LUT.

#### *k*-Means Cluster Analysis

A hyperspectral stack of SRS images across the high-wavenumber region (2800–3050 cm^–1^) was imported into ImageJ and an average intensity projection was generated. Multivariate analysis of the SRS spectral data was performed by KMCA as described in McRae et al.^33^ using a plugin for ImageJ. Cell segmentation of LDs by KMCA facilitated analysis at different wavenumbers throughout the image stack. Upon segmentation of the LD cluster, the image was converted into a binary mask, from which the SRS intensity at the LD regions was created.

### Statistical Analysis

Statistical analyses and data plotting were performed using GraphPad PRISM software v9.3.1 (GraphPad Software Inc., San Diego, CA, USA).

## Supporting Information Available

Experimental data and procedures for Raman spectroscopy and SRS imaging reported. Representative workflow schematic of KMCA, Raman spectral images and segmentation of cellular components (PDF)

## Abbreviations

ACC: acetyl-coA carboxylase
AR: androgen receptor
ATT: androgen targeted therapy
CRPC: castration-resistant prostate cancer
DMEM: Dulbecco’s modified Eagle’s medium
DNL: de novo lipid synthesis
EOM: electro-optic-modulator
FAO: fatty acid oxidation
FAS: fatty acid synthase
FBS: heat-inactivated foetal bovine serum
LDs: lipid droplets
LNCaP: lymph node carcinoma of the prostate
MS: mass spectrometry
OPO: optical parametric oscillator
PC3: human prostate adenocarcinoma
PCa: prostate cancer
PCA: principal component analysis
PNT2: human normal immortalized prostate epithelium
RPMI: Roswell Park Memorial Institute Medium
SCD-1: stearoyl-coA desaturase-1
SREBP: sterol regulatory-element binding protein
SRS: stimulated Raman scattering
TEM: transmission electron microscopy
TOFA: 5-(tetradecyloxy)-2-furoic acid
