# Supplementary material for: Label-Free Imaging of Lipid Droplets in Prostate Cells Using Stimulated Raman Scattering Microscopy and Multivariate Analysis
Source: Anal Chem. 2022 Jun 14;94(25):8899–908. doi: 10.1021/acs.analchem.2c00236 (PMC9244870; doi:10.1021/acs.analchem.2c00236)
Supplement: Supplementary file 1 — ac2c00236_si_001.pdf [file ac2c00236_si_001.pdf]

Supporting Information for

Label-free imaging of lipid droplets in prostate cells using stimulated Raman scattering microscopy and multivariate analysis.

Ewan W. Hislop, William J. Tipping, Karen Faulds,* and Duncan Graham*

Centre for Molecular Nanometrology, WestCHEM, Department of Pure and Applied Chemistry, Technology and Innovation Centre, University of Strathclyde, 99 George Street, Glasgow, G1 1RD, U.K.

*Email: duncan.graham@strath.ac.uk; karen.faulds@strath.ac.uk

Table of Contents

1. Experimental procedures ... 3
1.1 Raman spectroscopy ... 3
1.2 Sample preparation for time-lapse SRS microscopy ... 3
1.3 Data processing ... 3
2. Supplementary figures ... 4
Figure S1 Workflow schematic of K-means cluster analysis ... 4
Figure S2 Biological characterisation of live-cell phenotypes by spontaneous and stimulated Raman microscopy. ... 5
Figure S3 K-means cluster segmentation of nuclei and LD components. ... 6
Figure S4 Characterisation of live LNCaP cells by stimulated Raman scattering microscopy. ... 7
Figure S5 Characterisation of live PC3 cells by stimulated Raman scattering microscopy. ... 8
Figure S6 Characterisation of live PNT2 cells by stimulated Raman scattering microscopy. ... 9
Figure S7 Ratiometric analysis of fixed cells by spontaneous Raman imaging. ... 10
3. References ... 11

1. Experimental procedures

1.1 Raman spectroscopy

All Raman spectra were acquired on a Renishaw inVia Raman microscope equipped with a 532 nm Nd:YAG laser giving a maximum power of 50 mW, using a 1800 l/mm grating and a Leica 50x/NA 0.75 N PLAN EPI objective at room temperature. Prior to spectral acquisition, calibration of the laser was performed using the internal silicon standards at 520.5 cm^{-1}. Raman imaging: PC3, LNCaP and PNT2 cells were seeded with a density of 1 x 10^{6} cells/mL onto high precision coverslips (#1.5H Thickness, 22 x 22 mm, Thorlabs) in 6-well culture dishes (Costar®) with 2 mL of their respective media for 24 h prior to treatment. Cells were treated with TOFA (5-20 μM) from a 20 mM stock solution in DMSO and incubated at 37 °C and 5% CO_2 for the indicated time. Prior to imaging, the dishes were aspirated and washed with PBS (2 × 2 mL), fixed with paraformaldehyde (4% in PBS, 15 min at room temperature). Cells were imaged using λex = 532 nm, with a step size of 1 μm in x and y, 0.5 s acquisition time, 50% laser power (c.a.18 mW laser power) and a spectral centre of 3000 cm^{-1}.

1.2 Sample preparation for time-lapse SRS microscopy

Harvested cells (PC3, LNCaP & PNT2) were seeded with a density of 1 x 10^{6} cells onto high precision coverslips (#1.5H Thickness, 22 x 22 mm, Thorlabs) in 6-well culture dishes (Costar®) with 2 mL of their respective media and incubated at 37 °C and 5% CO_2 for 24 h prior to treatment. From a 20 mM stock solution in DMSO cells were treated with TOFA (5 μM) in media and incubated at 37 °C and 5% CO_2 for the indicated time. Control cells were concomitantly treated with DMSO at an equivalent rate in the respective media (0.05% DMSO v/v). Throughout the course of treatment at the indicated timepoints, cells were analysed by SRS microscopy. Control and drug-treated cells were washed with PBS (2×2 mL). Coverslips were then mounted to glass microscope slides with a PBS boundary between the glass layers prior for live-cell imaging.

1.3 Data processing

Raman maps. Raman cell spectra were pre-processed in WiRE 4.4 software: cosmic ray removal, noise filtering and baseline subtraction. Output files were saved in .wdf, .spc and .txt formats. An in-house MATLAB® script (Ref. 16) used to segment cell regions based on the spectral intensity and normalised between 0-1. False-colour assignments for the cell regions were created based on the following peak intensity ratio 2851 cm^{-1}/(2851 cm^{-1} + 2933 cm^{-1}) and average spectra were generated, as reported in ESI Figure 7.

SRS images. A typical field of view (~20 cells, 40x objective lens) was acquired for each timepoint and condition (drug *vs.* control). Three replicate images were captured at different locations across the monolayer of cells at distinctive Raman shifts: 2930 cm^{-1} and 2851 cm^{-1} corresponding to cellular biomolecules of interest. All images were assigned relevant false-colours and scale bars using Image J software. Consistent brightness/contrast settings were applied throughout as detailed in the figure legends.

Lipid droplet analysis. A binary image of the 2930 cm^{-1} (CH_3, protein) signal was used for threshold analysis and together with a watershed function in Image J, was used to segment regions-of-interest (ROI) analogous with the area of each cell image. Lipid droplet distribution across the cell was analysed by thresholding pixels with higher Raman scattering intensity in the 2851 cm^{-1} images corresponding to lipid-rich droplets. The percentage area of lipid droplets per cell was calculated using measured values in Image J in the equation:

$$\% \, Area \; of \; LD \; per \; cell \; = \; (Thresholded \; ROI \; in \; 2851 \; cm^{-1} \; / Total \; ROI \; cellular \; area \;) \times 100.$$

2. Supplementary figures

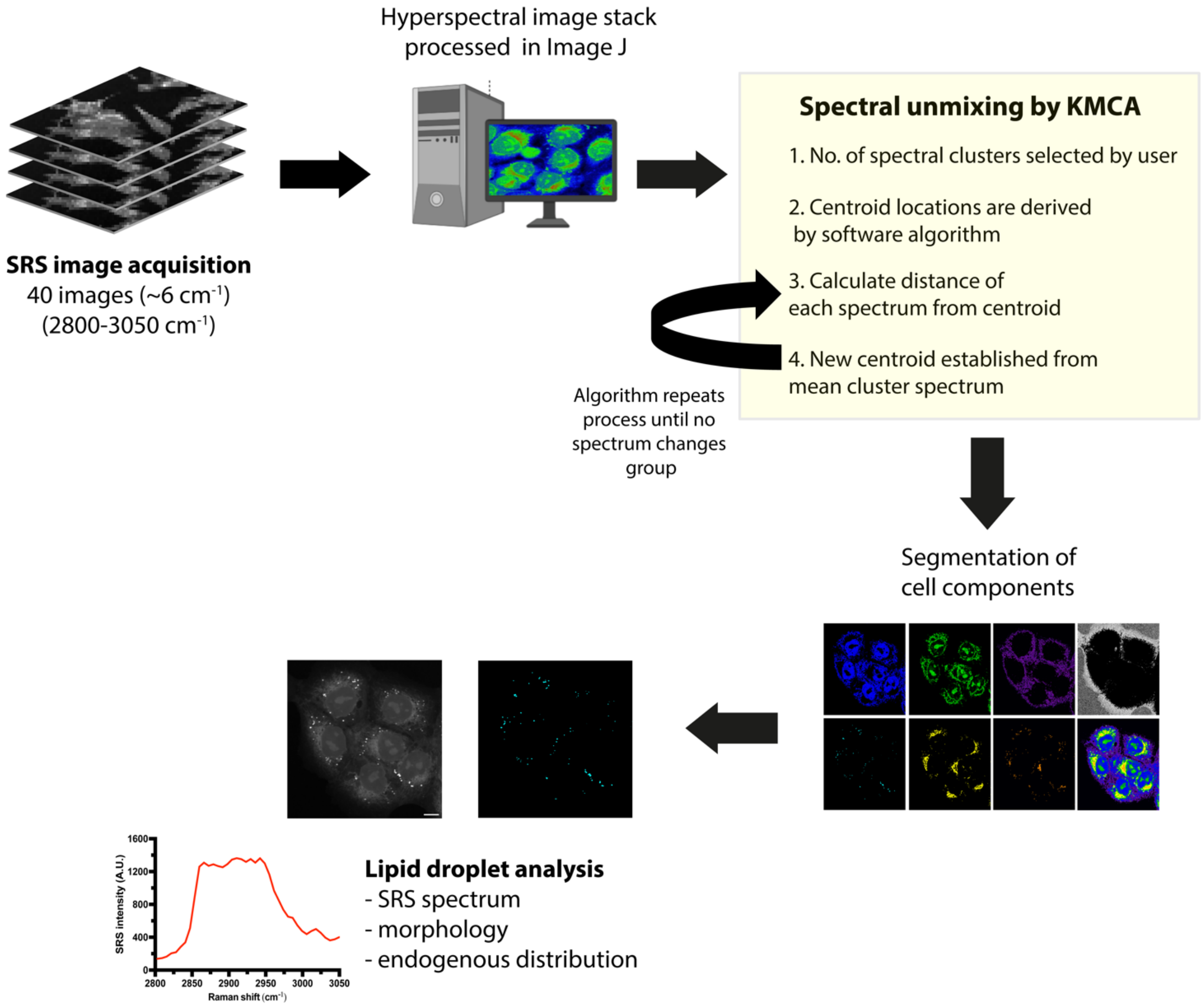

Figure S1 Workflow schematic of K-means cluster analysis .

A illustrative description of K-means cluster analysis and cell component segmentation from SRS image acquisition to lipid droplet data processing steps.[1]

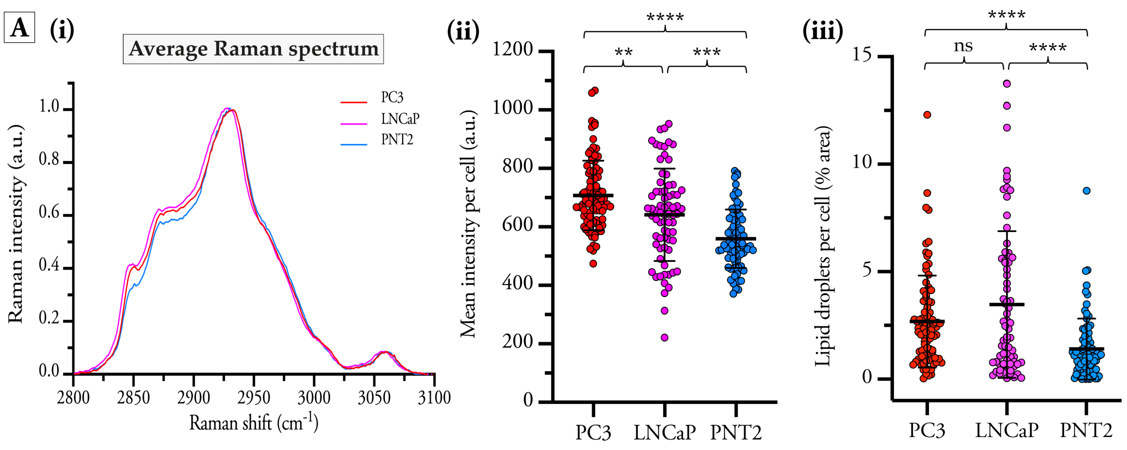

Figure S2 Biological characterisation of live-cell phenotypes by spontaneous and stimulated Raman microscopy.

A (i) The average spectral response of PCa and normal prostate by spontaneous Raman spectroscopy in the HWN region (2800 - 3100 cm^{-1}). Scatter plot of (ii) endogenous lipid biomolecules at 2851cm^{-1} and (iii) LD levels between control cell lines acquired by stimulated Raman spectroscopy. Lipid metabolism is upregulated in PCa cells, demonstrated by an increase in peak intensities at 2851cm^{-1} and 2880 cm^{-1}. Student's t-test was employed to assess significance between the controls (**** $p \leq 0.0001$).

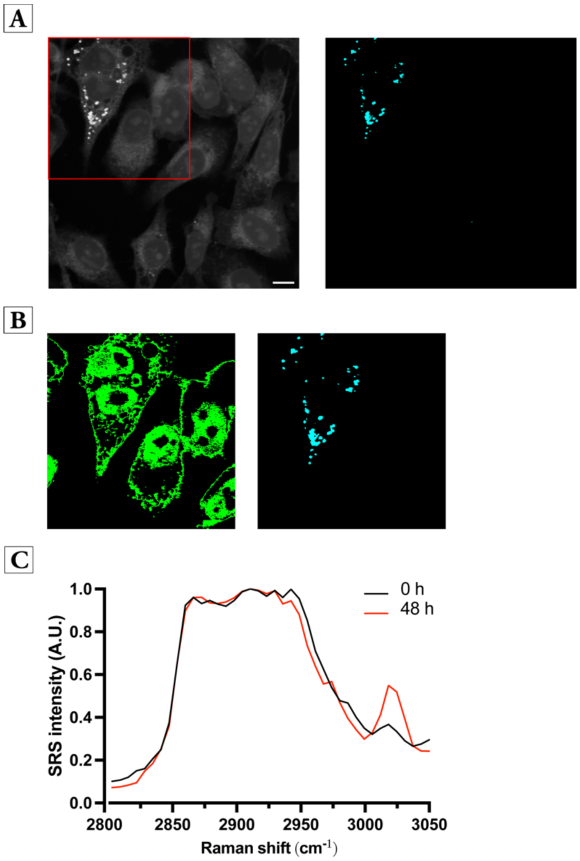

Figure S3 K-means cluster segmentation of nuclei and LD components.

A Hyperspectral SRS image of PC3 cells exposed to TOFA for 48h, highlighting a multinucleated cell phenotype with high LD accumulation. **B** KMCA enabled multiple nuclei (green) and dense LD areas (cyan) to be segmented from the cell. **C** SRS spectral analysis of LD components after 48h demonstrated an increase in associated TAG at 3015 cm^{-1}.

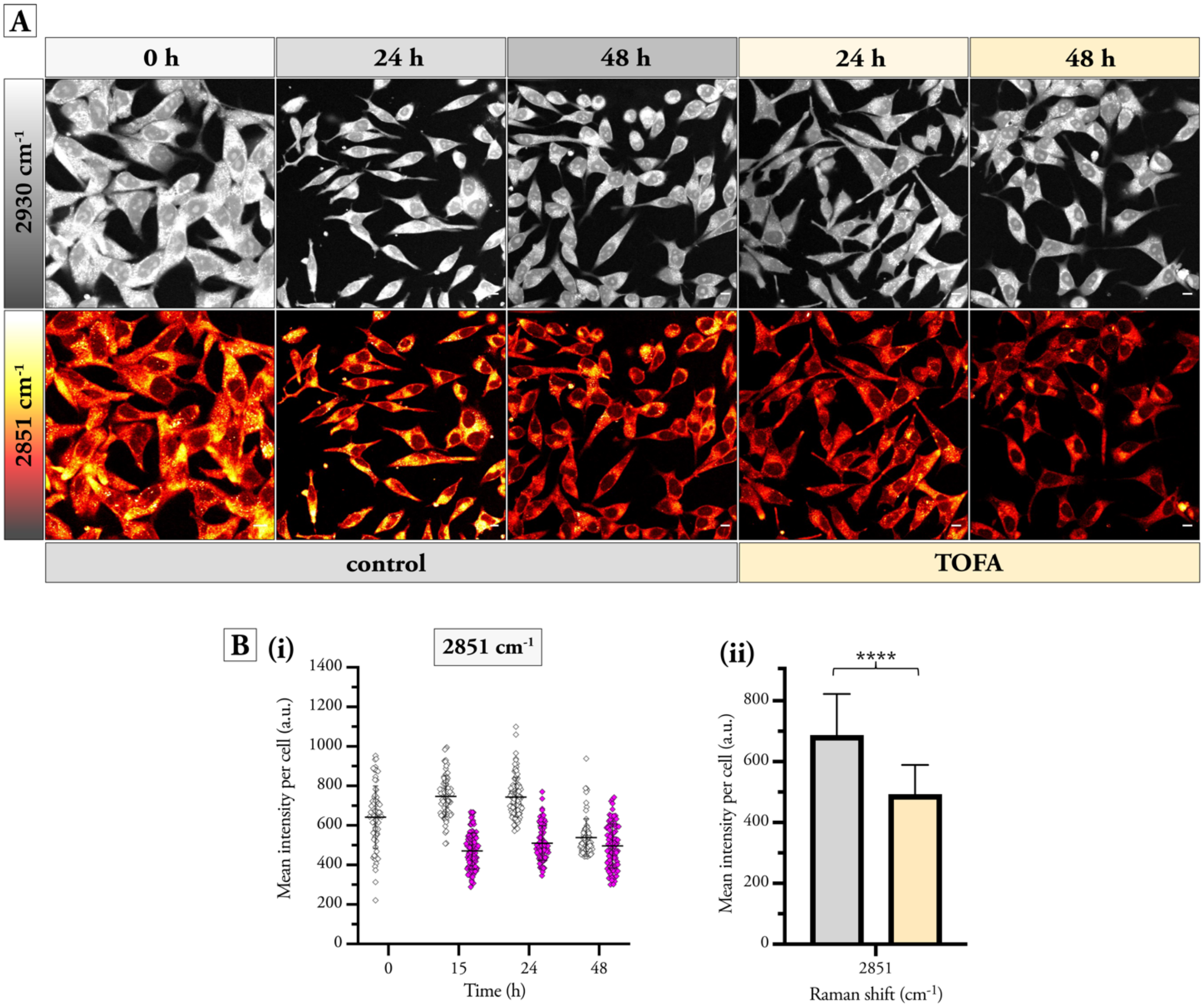

Figure S4 Characterisation of live LNCaP cells by stimulated Raman scattering microscopy.

A LNCaP cells were imaged live using SRS microscopy at the following: 2930 cm^{-1} (CH_3, proteins) and 2851 cm^{-1} (CH_2, lipids). Images were acquired at a frame size of 512 × 512 pixels, using a 48 µs pixel dwell time with false colours applied to different detection wavenumbers. A sampling population of >100 cells per time point were assessed across three repeats to evaluate TOFA (5 µM) treatment at the indicated timepoints. Look-up table (LUT): 0–2500 a.u., scale bars: 10 µm. **B** (i)Quantification of the mean SRS intensity at 2851 cm^{-1} (magenta) per cell. Two-way ANOVA was performed to compare the change in mean intensity vs. the effect of treatment against time (**** $p \leq 0.0001$). (ii) A bar chart summarises the changes to the mean intensities at 2851 cm^{-1} per cell after 48 h in the presence of DMSO (grey) or TOFA (yellow) and a student's t-test was performed to compare mean values (**** $p \leq 0.0001$).

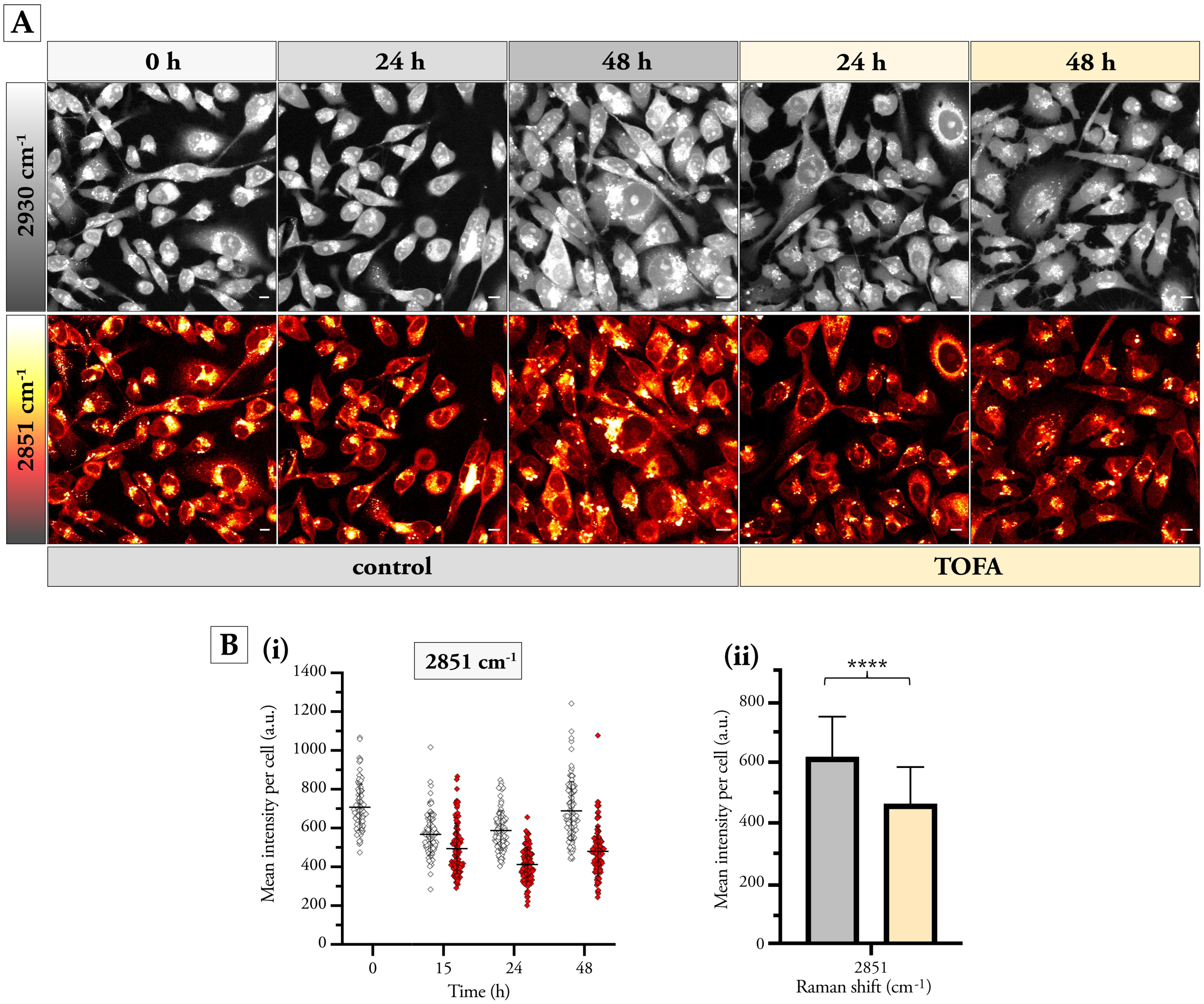

Figure S5 Characterisation of live PC3 cells by stimulated Raman scattering microscopy.

A PC3 cells were imaged live using SRS microscopy at the following: 2930 cm^{-1} (CH_3, proteins) and 2851 cm^{-1} (CH_2, lipids). Images were acquired at a frame size of 512 × 512 pixels, using a 48 µs pixel dwell time with false colours applied to different detection wavenumbers. A sampling population of >100 cells per time point were assessed across three repeats to evaluate TOFA (5 µM) treatment at the indicated timepoints. Look-up table (LUT): 0–2500 a.u., scale bars: 10 µm. **B** (i) Quantification of the mean SRS intensity at 2851 cm^{-1} (red) per cell. Two-way ANOVA was performed to compare the change in mean intensity *vs.* the effect of treatment against time (**** $p \leq 0.0001$). (ii) A bar chart summarises the changes to the mean intensities at 2851 cm^{-1} after 48 h in the presence of DMSO (grey) or TOFA (yellow)and a student's t-test was performed to compare mean values (**** $p \leq 0.0001$).

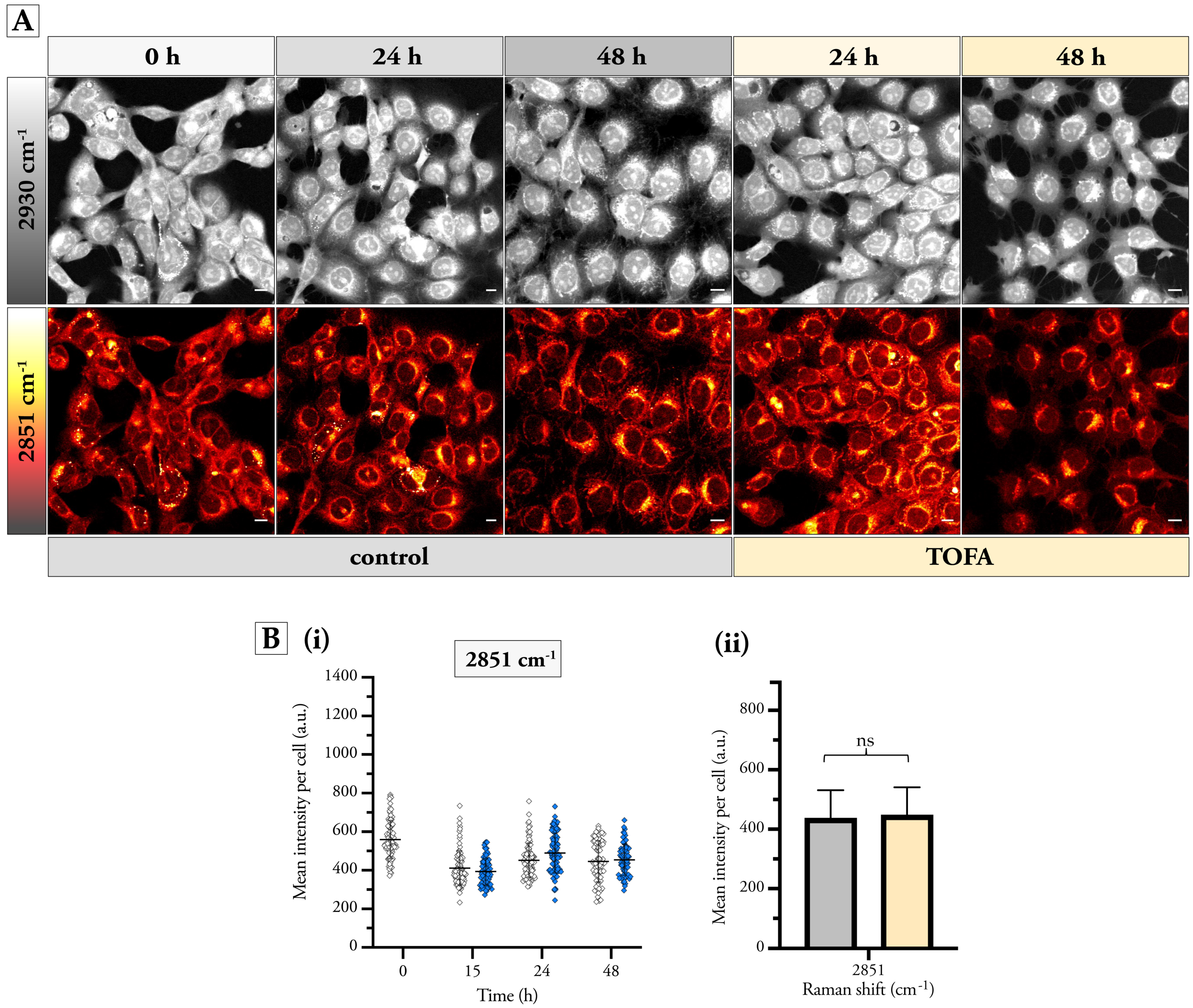

Figure S6 Characterisation of live PNT2 cells by stimulated Raman scattering microscopy.

A PNT2 cells were imaged live using SRS microscopy at the following: 2930 cm^{-1} (CH_3, proteins) and 2851 cm^{-1} (CH_2, lipids). Images were acquired at a frame size of 512 × 512 pixels, using a 48 µs pixel dwell time with false colours applied to different detection wavenumbers. A sampling population of >100 cells per time point were assessed across three repeats to evaluate TOFA (5 µM) treatment at the indicated timepoints. Look-up table (LUT): 0–2500 a.u., scale bars: 10 µm. **B** (i) Quantification of the mean SRS intensity at 2851 cm^{-1} (blue) per cell. Two-way ANOVA was performed to compare the change in mean intensity *vs.* the effect of treatment against time (ii) A bar chart summarises the changes to the mean intensities at 2851 cm^{-1} after 48 h in the presence of DMSO (grey) or TOFA (yellow) and a student's t-test was performed to compare mean values (n.s. not significant).

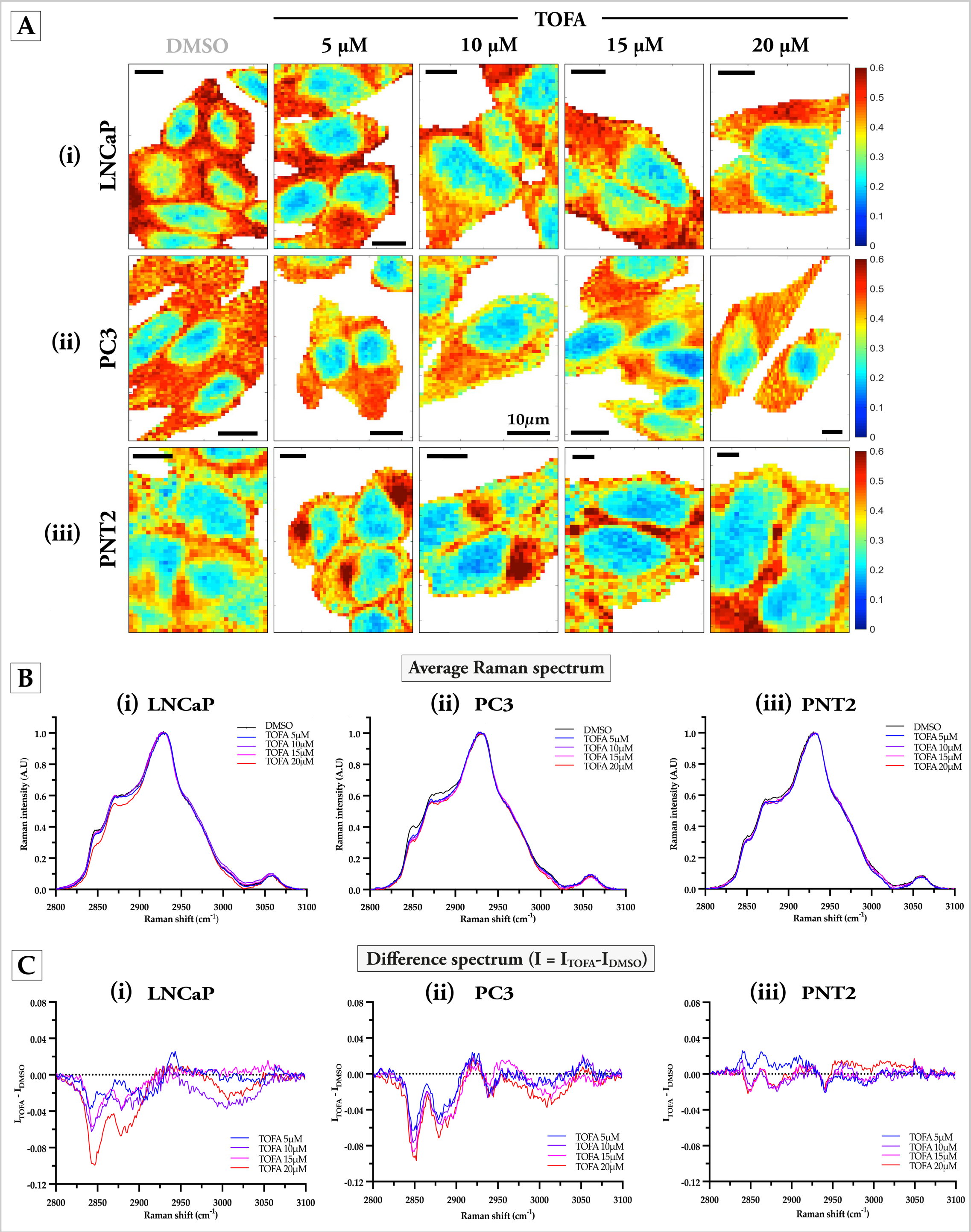

Figure S7 Ratiometric analysis of fixed cells by spontaneous Raman imaging.

(i) LNCaP, (ii) PC3 and (iii) PNT2 cell lines were treated with DMSO (control) or TOFA (5 μM - 20 μM) for 48 h. Raman images acquired from fixed cells using 532 nm laser excitation for 0.5 s using a 50× objective lens (~16 mW) with a 1 μm pixel size. **A** Ratiometric Raman images representing the ratio of peak intensities 2851 cm^{-1} / (2851 cm^{-1} + 2930 cm^{-1}). Scale bars: 10 μm. **B** Average Raman spectra normalised to the intensity of the 2930 cm^{-1} (CH_3 symmetric stretch) from the cell maps presented in A. **C** A line plot demonstrates the difference in Raman intensity of cells treated with TOFA minus the Raman intensity of the DMSO control ($I = I_{TOFA} - I_{DMSO}$). Values below 0 indicate the Raman intensity at that wavenumber is lower in the TOFA treated sample than in the DMSO control and *vice versa*.

3. References

1 Byrne, H. J.; Knief, P.; Keating, M. E.; Bonnier, F., Spectral pre and post processing for infrared and Raman spectroscopy of biological tissues and cells. *Chem Soc Rev* **2016,** *45* (7), 1865-1878.
